# Past and Future Exceedances of Nitrogen Critical Loads in Europe

**DOI:** 10.1100/tsw.2001.302

**Published:** 2001-11-14

**Authors:** Maximilian Posch, Jean-Paul Hettelingh, Petra Mayerhofer

**Affiliations:** ^1^ National Institute for Public Health and the Environment (RIVM), P.O.Box 1, NL-3720 BA Bilthoven, The Netherlands; ^2^ Center for Environmental Systems Research, University of Kassel, Kurt-Wolters-Str. 3, D-34109 Kassel, Germany

**Keywords:** critical loads, nitrogen, exceedance, Europe, climate change

## Abstract

Critical loads of acidity and nutrient nitrogen — simple measures of the sensitivity of ecosystems to deposition — have been widely used for setting emission reduction targets in Europe. In contrast to sulfur, the emissions of nitrogen compounds remain high in the future. This is also true for the exceedances of critical loads until 2010. Looking further into the future, climate change is likely to influence ecosystem sensitivity, and thus critical loads. It is shown that higher temperatures, changed precipitation patterns, and modified net primary production mainly increase critical loads, except in mountainous and arid regions. Using consistent scenarios of climate change and air pollution from a recently completed European study (AIR-CLIM), it is shown that the exceedances in 2100 of the critical loads are declining in comparison to 2010. However, exceedances of critical loads of nutrient nitrogen remain substantial, even under the most stringent scenario. This confirms the increasing role nitrogen plays in environmental problems in comparison to sulfur. Thus research should focus on the effects of nitrogen in the environment, especially under conditions of climate change, to support nitrogen-emission mitigating policies. This not only reduces acidification and eutrophication, but also helps curb the formation of tropospheric ozone.

## INTRODUCTION

Nitrogen in its various forms has a history as a pollutant spanning more than 4 decades. In the 1960s and 1970s, it was the excessive discharge of nitrogen and phosphorus which led to widespread eutrophication of surface waters in many parts of the (developed) world. While this problem could be mostly solved on a local or national scale, the acidification of surface waters (and later also forest soils), which slowly caught the attention of the public during the 1970s, turned out to be of a truly transboundary nature. Airborne acidifying substances can be transported hundreds and even thousands of kilometers before being deposited in areas not affected by local human activities. This insight led to the signing of the UN/ECE Convention on Long-Range Transboundary Air Pollution (LRTAP) in 1979, in which the signatories (i.e., the European, including the Soviet Union, and North American countries) pledged "to limit and ... gradually reduce ... long-range transboundary air pollution" (Art. 2). Since until recently the emissions of sulfur far exceeded those of nitrogen compounds, the first Protocol to the LRTAP Convention on emission reductions (signed in 1985) dealt with sulfur only. However, in 1988, a protocol was signed which obliged the parties not to increase their national $NO_x$ emissions. Although the obligations themselves were not very demanding, in the same protocol the parties agreed to establish critical loads and use them in negotiations on further emission reductions.

Critical loads of sulfur acidity were the first ones to be computed and mapped on a European scale, and they were used in the negotiations of the so-called Second Sulfur Protocol, signed in 1994. By then it had been recognized that the problem of acidification demands the simultaneous consideration of sulfur and nitrogen compounds. Considering also the role of nitrogen as a nutrient and as a precursor in the formation of tropospheric ozone, the scientific basis and methods for calculating critical loads of sulfur and nitrogen acidity and of eutrophication, as well as critical levels for ozone, were established, and they were used in the negotiations of a “multipollutant, multieffects” protocol which was signed in 1999.

Critical loads are, by definition, steady-state quantities, i.e., it is assumed, *inter alia*, that the climatic variables used in their calculation remain constant over time. Furthermore, the long-range transport models used to derive deposition fields for (future) exceedance calculations assume that the (average) source-receptor relationships will not change in the future. Considering, however, that there is a scientific consensus that the Earth’s climate will change due to emissions of greenhouse gases, it seems prudent to investigate the influence of a changing climate on critical loads and the long-range transport of pollutants in order to assess the robustness of current emission reduction policies.

Not only does climate change influence critical loads and their exceedances, but nitrogen plays an important role in global change itself[1]. Both ammonia and the oxides of nitrogen are greenhouse gases (directly or via the formation of tropospheric ozone), and thus their reduction leads to additional environmental benefits. Due to the strong link between carbon and nitrogen in terrestrial (and aquatic) ecosystems, any change in the amount of nitrogen deposited will have an impact on the pools and fluxes of carbon (organic matter), and thus influence the amount of carbon retained in soils. This, in turn, could influence the nitrogen processes in the soil and invalidate the assumptions on which critical load calculations are based.

In this paper, we first summarize the methods for calculating critical loads of acidity and nutrient nitrogen and present maps showing their magnitude and geographic distribution in Europe. In addition, we present and discuss maps of the temporal development of their exceedance between 1960 and 2010. Next, we present some results of a recently completed study (AIR-CLIM), which investigated the influence of climate change on critical loads and their exceedances under various scenarios of global change, derived from the recent IPCC scenarios. In a final section, we present some conclusions and a plea for closer collaboration between scientists working in the field of nitrogen processes and policy makers concerned with emission reduction measures.

## CRITICAL LOADS AND THEIR EXCEEDANCES

The concept of a critical load is based on the assumption that it is possible to determine an (ecological) threshold above which deposition leads to detrimental effects. Critical loads of (sulfur) acidity were first introduced in 1983 in Canada[2]. With strong support from the Nordic countries since the mid-1980s, the concept has been further developed in Europe. A critical load has been defined as “the quantitative estimate of an exposure to one or more pollutants below which significant harmful effects on specified sensitive elements of the environment do not occur according to present knowledge”[3]. Starting from this general definition, methodologies have been developed during the 1990s for calculating and mapping critical loads in Europe under the auspices of the UN/ECE LRTAP Convention, and they are documented in a so-called “Mapping Manual”[4].

Critical loads of sulfur and nitrogen can be calculated for various receptors (forest soils, surface waters, semi-natural vegetation) using different methods (empirical, steady-state, or dynamic models). In order to give an impression of the kind of data needed, below we summarize the equations for computing acidity and nutrient nitrogen critical loads for forest soils derived from the steady-state Simple Mass Balance (SMB) model, the most widely used method for calculating critical loads in Europe.

The starting point of the SMB model is the charge balance of the major ions in the soil solution leaving the rooting zone of the forest soil. Both sulfur and nitrogen contribute to the acidification of soils, and there is no unique critical load of sulfur or nitrogen with respect to acidification. Introducing several simplifying assumptions, the maximum critical load of sulfur is derived as:

$$CL_{max}(S) = BC_{dep}^* + BC_w - Bc_u - ANC_{le(crit)} \qquad (1)$$

where BC stands for the sum of base cations (BC = Bc + Na = Ca + Mg + K + Na) and the subscripts w and u stand for weathering and (net) growth uptake, respectively. The * refers to sea-salt-corrected BC deposition, assuming all chloride is coming from sea spray. $ANC_{le(crit)}$ is the critical leaching of acid neutralizing capacity, derived from a chosen critical chemical limit (e.g., the [Al]/[Bc] ratio in the soil solution) which links chemistry with biological impacts. $CL_{max}(S)$ is sometimes also referred to as the critical load of (potential) acidity.

As long as the deposition of N, $N_{dep}$, stays below the minimum critical load of nitrogen, i.e.,

$$N_{dep} \leq CL_{min}(N) = N_i + N_u \qquad (2)$$

all deposited N is consumed by sinks of N (i.e., immobilization, $N_i$, net growth uptake, $N_u$, and denitrification), which is characterized by the fraction $f_{de}$ of the net input of N which is denitrified. The maximum critical load of nitrogen (in case of no S deposition) is given by:

$$CL_{max}(N) = CL_{min}(N) + CL_{max}(S)/(1–f_{de}) \qquad (3)$$

The three critical loads from Eq. 1–3 define the so-called critical load function of an ecosystem (Fig. 1); and every combination of $N_{dep}$ and $S_{dep}$ lying on or below that function does not cause “harmful effects” (i.e., does not result in critical load exceedance).

Excess nitrogen deposition contributes not only to acidification, but can also lead to the eutrophication of soils, inducing, for example, unwanted changes in species composition. Thus a critical load of nutrient nitrogen has been derived as:

$$CL_{nut}(N) = CL_{min}(N) + Q[N]_{acc}/(1–f_{de}) \qquad (4)$$

where Q is the water leaving the root zone (estimated as precipitation minus evapotranspiration) and $[N]_{acc}$ is a maximum acceptable N concentration in the soil solution. The derivation of the above critical load quantities from basic equations (charge and mass balances) and a discussion of the assumptions and simplifications involved can be found elsewhere[4,5].

For the scientific support of the negotiations of the recent protocols, critical loads data have been computed by the indi-

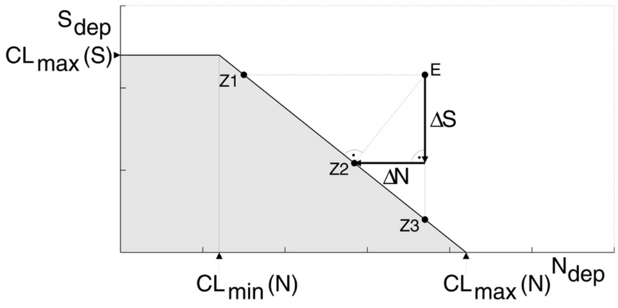

**FIGURE 1.** Example of a critical load function for acidifying nitrogen and sulfur defined by the three quantities $CL_{max}(S)$, $CL_{min}(N)$, and $CL_{max}(N)$. The gray-shaded area below the function represents depositions of N and S for which there is nonexceedance of acidity-critical loads. Also indicated is the way in which the exceedance (AAE) is calculated (see below).

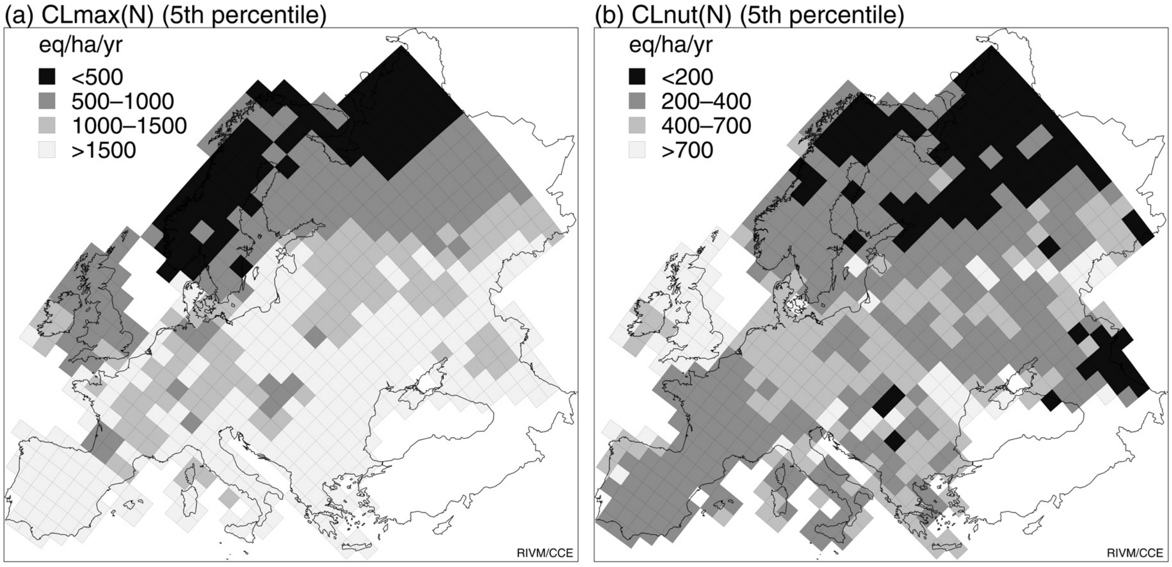

**FIGURE 2.** Fifth percentile of the maximum critical load of nitrogen (left) and the critical load of nutrient N (right) in the EMEP 150 × 150 $km^2$ deposition grid covering Europe (1000 eq/ha = 1.4 $gN/m^2$).

vidual European countries for a variety of receptors (forest soils, lakes, semi-natural vegetation) and submitted to the Coordination Center for Effects (CCE), which is part of the Mapping Programme under the LRTAP Convention. The CCE collates those national critical loads data and produces European maps and databases which are then used in the integrated assessment of emission reduction strategies[6]. In Fig. 2, maps of the 5th percentile of the maximum critical load of nitrogen (Eq. 3) and the critical load of nutrient N (Eq. 4) are displayed. It can be seen that (1) $CL_{nut}(N)$ is much smaller than $CL_{max}(N)$ and (2) the most sensitive ecosystems are concentrated in northern Europe.

To assess the risk of ecosystem damage due to a given scenario, critical loads are compared with the deposition of sulfur and nitrogen. Within the LRTAP Convention, deposition fields are computed with the source-receptor matrices (SRMs) derived from the EMEP long-range atmospheric transport model[7]. The SRMs derived for the meteorological years 1985 through 1996 were averaged to minimize the effects of interannual variability. With the aid of these SRMs and the sulfur and nitrogen ($NO_x$ and $NH_3$) emissions of the European countries, the respective depositions in every grid cell are computed.

If depositions are greater than critical loads, we say the critical loads are exceeded. While in the case of a single pollutant the exceedance can be defined in an obvious manner, e.g., $Ex(N_{dep}) = N_{dep}-CL_{nut}(N)$, there is no unique exceedance (i.e., amount of deposition to be reduced to reach nonexceedance) in the case of acidifying N and S. In Fig. 1, let the point E denote the depositions of N and S. Reducing $N_{dep}$ substantially, one reaches the point Z1 and thus nonexceedance without reducing $S_{dep}$; on the other hand, one can reach nonexceedance by only reducing $S_{dep}$ (by a smaller amount) until reaching Z3. Intuitively, the reduction required in N and S deposition to reach point Z2 seems a good measure for exceedance. Therefore, we *define* the exceedance for a given pair of depositions ($N_{dep}$,$S_{dep}$) as the sum of the N and S deposition reduction required to reach the critical load function by the "shortest" path, i.e., $Ex(N_{dep},S_{dep}) = \Delta N + \Delta S$ (see Fig. 1).

For an assessment, all critical load functions within a grid cell have to be considered, and each ecosystem contributes with its area (weight) $A_i$, i = 1,...,n (n = number of ecosystems in the grid cell). Let $Ex_i$ be exceedances as defined above, then we define the average accumulated exceedance (AAE) as the area-weighed average of individual exceedances in a grid cell as:

$$AAE = (A_1Ex_1+...+A_nEx_n)/(A_1+...+A_n) \qquad (5)$$

AAEs have turned out to be robust indicators of critical load exceedances in the integrated assessment work leading to the 1999 Protocol to the LRTAP Convention. In Fig. 3, the temporal development (1960–2010) of the average accumulated exceedances is shown for acidity and nutrient nitrogen critical loads. The depositions of N and S in 2010 assume the full implementation of the LRTAP Protocols.

As can be seen from Fig. 3, exceedances of $CL_{nut}(N)$ are catching up with acidity exceedances between 1960 and 1990, and in 2010, after the implementation of the LRTAP Protocols, the exceedances of acidity critical loads will have almost vanished, whereas there are still substantial areas where $CL_{nut}(N)$ is exceeded. From this, it is obvious that the role of sulfur will further diminish on a regional scale, and nitrogen species will become the dominant transboundary air pollutants in Europe.

## CRITICAL LOADS AND CLIMATE CHANGE

By definition, critical loads are steady-state quantities and do not change over time, unless new data or methods require their re-evaluation. Since climate-related parameters enter the critical load calculations, any change in climate will influence critical loads. As part of an EU-funded project, AIR-CLIM, the impact of climate change (policies) on critical loads has been analyzed, providing an indication of the robustness of current European air pollution abatement policies under various climate change scenarios[8].

Eight scenarios for different combinations of future greenhouse gas (GHG) and sulfur and nitrogen emissions, covering the years 1990–2100, were developed during the AIR-CLIM project (see Table 1). Four each are based on the A1 and B1 scenario "story line" underlying the recently published scenarios of the IPCC[9]. The temporal development of the total European emissions of nitrogen is depicted in Fig. 4. It shows that the emissions in the A1-world are in generally higher than those in the B1-world, and that nitrogen emissions are going to surpass sulfur emissions in the not-too-distant future in all scenarios.

Critical loads depend on climatic variables in several ways. Base cation weathering is a function of temperature, T:

$$BC_w(T) = BC_w(T_0)\exp(A/T_0-A/T) \qquad (6)$$

where $T_0$ is the reference temperature and A = 3600K. The critical ANC leaching in $CL_{max}(S)$ (Eq. 1), as well as $CL_{nut}(N)$ (Eq. 4), depend on the runoff Q which, in turn, depends on precipitation and temperature needed to compute evapotranspiration. This is done with the same procedure[10] as used in the IMAGE

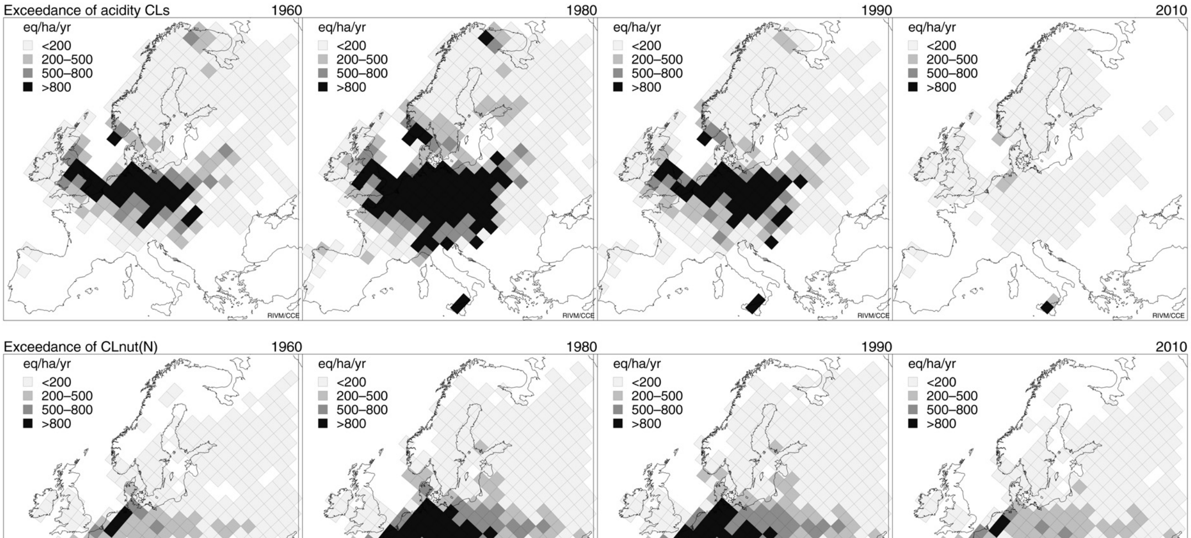

**FIGURE 3.** Temporal development (1960–2010) of the average accumulated exceedance (AAE) of acidity critical loads (top row) and critical loads of nutrient nitrogen (bottom). White areas indicate nonexceedance (or lack of data).

**TABLE 1**
**Overview of AIR-CLIM Scenarios[8]**

| Scenario | Start Scenario | Greenhouse Gas Policies | $SO_2/NO_x$ Policies |
|---|---|---|---|
| A1-P | A1 | None | Present policies |
| A1-A | A1 | None | Advanced policies |
| A1-550-P | A1 | To achieve 550 ppm stabilization | Present policies |
| A1-550-A | A1 | To achieve 550 ppm stabilization | Advanced policies |
| B1-P | B1 | None | Present policies |
| B1-A | B1 | None | Advanced policies |
| B1-450-P | B1 | To achieve 450 ppm stabilization | Present policies |
| B1-450-A | B1 | To achieve 450 ppm stabilization | Advanced policies |

*Note:* Present air pollution policies (-P): compliance with 1999 LRTAP Protocol and equivalent measures in regions without policies up to now; advanced air pollution policies (-A): further reductions after compliance with 1999 LRTAP Protocol.

model[11]. Furthermore, nutrient uptake by forests depends on climate variables, and they have been scaled with the changing net primary production (NPP) predicted by the IMAGE model.

For the AIR-CLIM assessment, the eight emission scenarios have been fed into the IMAGE global change model[11] to compute temperature and precipitation changes for the period 2000–2100. The "official" critical loads used in the protocol negotiations (Fig. 2) could not be used to assess the impact of climate change, since the input parameters to compute them were not available, and thus they could not be recomputed for climate-changed parameters. Thus a uniform European database[12], which is also used to compute critical loads for countries which do not submit national data, has been used to assess the influence of climate change on the critical load quantities in the AIR-CLIM project. Fig. 5 shows which changes in critical load of nutrient nitrogen can be expected between 2030 and 2100 under the A1-P scenario. A negative change (dark areas in Fig. 5) means that forest soils become more sensitive to the deposition of nitrogen. While increases in sensitivity — due to a decrease in runoff — are mostly found on the western Iberian peninsula and the mountainous regions (Alps), decreases in sensitivity are found throughout the northern parts of Europe, mostly due to an increase in nitrogen uptake.

An exceedance is a measure for the *amount* of deposition by which critical loads are exceeded, and the AAE defined in Eq. 5 is the average amount over the total ecosystem area in a grid cell. This has the disadvantage of not characterizing the *extent* of the problem, i.e., it says nothing about the ecosystem area that is exceeded within a single grid cell. Although there is a positive correlation between the amount and the extent of exceedance, the maps in Fig. 3 can be misleading, since a grid cell shown as "exceeded" can refer to a large area, such as in the Nordic countries, or to small patches of forest, such as in most parts of southern Europe.

It is always possible to uniquely determine whether a critical load is exceeded or not, without specifying the amount. Fig. 6 depicts the temporal development of the percentage of forest area for which critical loads of acidity and nutrient nitrogen are ex-

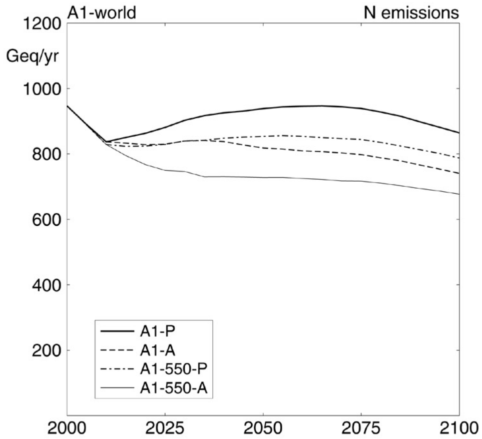

**FIGURE 4.** Temporal development (2000–2100) of the total European nitrogen emissions for the four scenarios each in the A1-world (left) and the B1-world (right) (1 Geq=14 ktN).

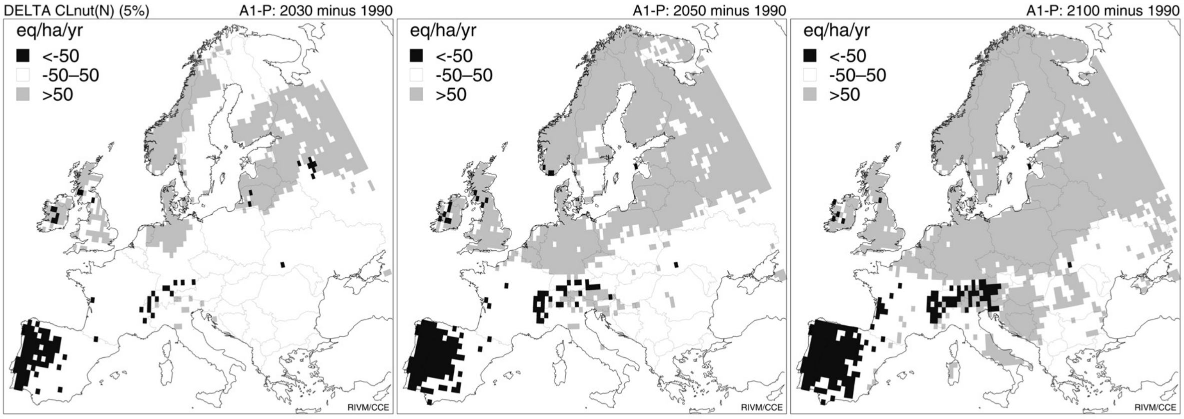

**FIGURE 5.** Change in the 5th percentile of $CL_{nut}(N)$ due to climate change according to the A1-P scenario in 2030 (left), 2050 (center), and 2100 (right).

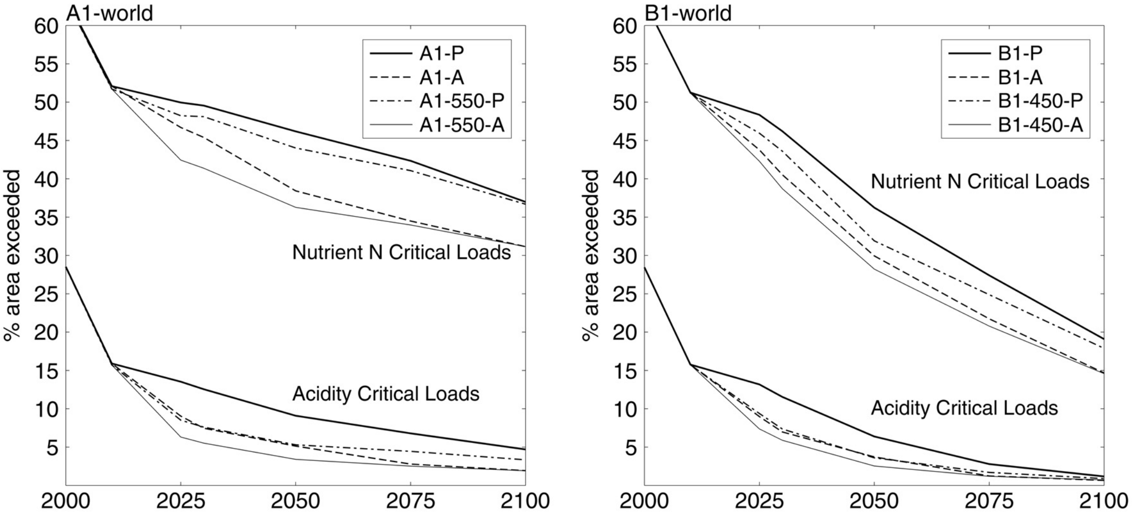

**FIGURE 6.** Temporal development of the percentage of forest area for which critical loads of acidity and nutrient nitrogen are exceeded for the four scenarios in the A1-world (left) and the corresponding four scenarios in the B1-world (right).

ceeded under the eight AIR-CLIM scenarios. The area for which critical loads are exceeded declines under all scenarios. The speed of decrease after 2010, however, differs between the two worlds, with larger decreases in the B1-world. In all cases, the A1-P and the B1-450-A scenarios are the least and most stringent one, respectively, with the other scenarios giving intermediate results. The most striking conclusion is that acidification (almost) ceases to be a problem, with exceedance percentages in 2100 between 4.7% (A1-P) and 0.7% (B1-450-A). Eutrophication by nitrogen, however, continues to be a widespread problem, even under the most stringent scenario, which brings the exceedance hardly down to 15% of the forest area. Thus nitrogen will be the main pollutant in need of future mitigation.

## DISCUSSION AND CONCLUSIONS

Nitrogen is the source of a multitude of environmental problems. Oxidized and reduced nitrogen causes acidification and eutrophication of soils and surface waters. Since these nitrogen compounds are transported over large distances, cost-effective reduction policies can only be arrived at through international collaboration. In Europe, within the framework of the UN/ECE LRTAP Convention, critical loads have been employed to characterize the vulnerability of ecosystems to the deposition of sulfur and nitrogen. In this paper, we show how the critical load exceedances (i.e., the increased risk of ecosystem damage) changes over 50 years (1960–2010). While sulfur, which was the main source of acidity until about 1990, will substantially decline with the implementation of the 1999 Protocol, agreed-upon nitrogen reductions are modest and thus exceedances of critical loads of nutrient N will remain substantial. In addition to acidification and eutrophication, nitrogen also contributes to the formation of ground-level ozone, which not only affects ecosystems but also human health. As a consequence, despite higher abatement costs, nitrogen will become the dominant pollutant on the agenda of the LRTAP Convention over the next years, if and when further mitigation measures have to be agreed.

But not only policy makers will increasingly have to deal with nitrogen in the context of acidification. Several assumptions in the calculation of critical loads are disputed and should be revisited before using critical loads for ever-more-costly reduction policies. For example, at present a large fraction of the deposited nitrogen is immobilized in the soil and does not leach from the root zone at most sites in Europe. Obviously, this cannot go on forever, and evidence is emerging that some forests in Europe (and North America) become nitrogen saturated[13]. Therefore, estimates of the long-term, sustainable immobilization of N have to be found and agreed upon to compute nitrogen critical loads for the review and possible revision of LRTAP protocols. More generally, the closer depositions get to critical loads, the more important it becomes to reduce — or at least quantify — the uncertainties inherent in critical load (and deposition) calculations. First steps in this direction under the LRTAP Convention have recently been undertaken[14].

Over the past years, public, and as a consequence scientific, attention to the environment has shifted to the issues of global change. Nitrogen in its various forms plays an important role in global change, both in the atmosphere and in terrestrial and aquatic ecosystems. Furthermore, there are potentially important linkages between regional air pollution and climate change. The AIR-CLIM project[8] addressed some of these issues and, with respect to critical loads, it was found that the sensitivity of forest ecosystems will decrease in most parts of Europe, especially in those where critical loads are low. In other words, current emission reduction policies are robust under a wide range of global change scenarios. However, it should be borne in mind that the impacts of climate change on critical loads are assessed with presently used models. Mostly due to a lack of knowledge, we did not consider the possible consequences that a change in climate could have on ecosystem processes (e.g., the nitrogen cycle) and which would require a reformulation of the critical load equations.

Two recommendations emerge from the work described in this paper:

1. Closer cooperation between scientists working on the impacts on terrestrial ecosystem of acidification/eutrophication on the one hand and of climate change on the other hand would help both communities.
2. In particular, policy makers should consider both problems — regional air pollution and global change — together, since major (monetary) benefits can be expected from harmonized mitigation policies.

Although it seems to be a win-win situation both for scientists and policy makers, many barriers, not only institutional, will have to be overcome to achieve this goal.

## ACKNOWLEDGMENT

The AIR-CLIM project was supported by the European Commission, Directorate General XII, within the EC Environment and Climate Research Programme (1994–1998), Contract No. ENV4-CT97-0449.

| | | |
|---|---|---|
| Received: | July | 21, 2001 |
| Revised: | October | 8, 2001 |
| Accepted: | October | 15, 2001 |
| Published: | November | 14, 2001 |

## BIOSKETCHES

**Maximilian Posch** holds a Ph.D. in Physics and a Masters degree in Mathematics from the Technical University of Vienna and worked from 1981 to 1989 at the International Institute for Applied Systems Analysis (IIASA) in Laxenburg (Austria) on the integrated assessment of acidification in Europe (the RAINS model). From 1990 to 1994 he worked at the Finnish Water and Environment Research Institute in Helsinki on the environmental impacts of acid deposition and agricultural practices. In 1995 he assumed (and currently holds) the position of Senior Researcher at the Coordination Center for Effects (CCE) at the Dutch National Institute for Public Health and the Environment (RIVM).

**Jean-Paul Hettelingh** holds a Ph.D. in Economics from the Free University in Amsterdam. From 1978 to 1985 he worked on environmental modeling and economics at the Institute for Environmental Studies of the Free University in Amsterdam. From 1985 to 1989 he was research scholar at the International Institute for Applied Systems Analysis (IIASA) in Austria contributing to the development of the RAINS model. From 1989 onwards he continued work on environmental modeling at the National Institute for Public Health and the Environment (RIVM) in The Netherlands. In 1991 he became head of the RIVM-Coordination Center for Effects (CCE) focusing on the assessment of environmental impacts of air pollution in support of European policy processes under the UN/ECE LRTAP Convention. Since 1997 he also holds a chair at the Centre for Environmental Science at the University of Leiden.

**Petra Mayerhofer** is a mechanical engineer with degrees from the University of Stuttgart, Germany, and the Georgia Institute of Technology in Atlanta, Georgia. From 1991 to 1998 she worked at the Institute of Energy Economics and the Rational Use of Energy (IER) at the University of Stuttgart. There, she did research on the impacts and external costs of energy systems. Since 1998 she has been involved in research on the interrelationship between climate change and air pollution at the Center for Environmental Systems Research at the University of Kassel, Germany.
